# Immune reaction and regulation in transplantation based on pluripotent stem cell technology

**DOI:** 10.1186/s41232-020-00125-8

**Published:** 2020-07-01

**Authors:** Ryo Otsuka, Haruka Wada, Tomoki Murata, Ken-ichiro Seino

**Affiliations:** Institute for Genetic Medicine, Hokkaido University, Kita-15, Nishi-7, Sapporo, Hokkaido 060-0815 Japan

**Keywords:** Pluripotent stem cell, Transplantation, Cell therapy, Immunological rejection, Immune regulation

## Abstract

The development of pluripotent stem cell (PSC)-based technologies provides us a new therapeutic approach that generates grafts for transplantation. In order to minimize the risk of immune reaction, the banking of induced pluripotent stem cells (iPSCs) from donors with homozygous human leukocyte antigen (HLA) haplotype is planned in Japan. Even though pre-stocked and safety validated HLA-homozygous iPSCs are selected, immunological rejection may potentially occur because the causes of rejection are not always due to HLA mismatches. A couple of studies concerning such immunological issues have reported that genetic ablation of HLA molecules from PSC combined with gene transduction of several immunoregulatory molecules may be effective in avoiding immunological rejection. Also, our research group has recently proposed a concept that attempts to regulate recipient immune system by PSC-derived immunoregulatory cells, which results in prolonged survival of the same PSC-derived allografts. PSC-based technologies enable us to choose a new therapeutic option; however, considering its safety from an immunological point of view should be of great importance for safe clinical translation of this technology.

## Background

The generation of induced pluripotent stem cells (iPSCs) boosted recent progress in the field of regenerative medicine. Since Takahashi and Yamanaka have reported their breakthrough discovery, pluripotent stem cells became familiar not only to researchers but also to the public [1, 2]. Their achievement opened the door for scientists to study organ or tissue regeneration, disease development, and drug discovery on the basis of iPSC-technology [3]. Under appropriate culture conditions, pluripotent stem cells (PSCs) can be directed their differentiation toward any cells or tissues. This fundamental feature of PSCs raised the possibility that PSCs, such as iPSCs and embryonic stem cells, can be an alternative source of transplant grafts. In 2014, the first clinical trial was carried out with patients suffering from age-related macular degeneration [4]. The team led by Professor Masayo Takahashi of Riken Center for Developmental Biology in Japan successfully transplanted retinal pigment epithelial cells induced from patient autologous iPSC. This first challenge triggered the emergence of following iPSC-based clinical trials [5], and nearly 50 trials can be found as active status in ClinicalTrials.gov (https://clinicaltrials.gov).

## Induced pluripotent stem cells and transplantation

Organ transplantation is often the only curative therapy for end-stage organ failure [6]; however, low donor availability is currently a severe problem in some cases [7]. One of the critical differences between iPSC and ESC is that iPSC can be induced from patients’ somatic cells. Theoretically, transplantation of autologous iPSC-derived cells may not cause immune rejection. So, this novel technology that reprograms mature cells into a pluripotent state appeared an innovative strategy to cope with graft shortage and immunological concerns accompanied by human leukocyte antigen (HLA)-mismatch. Although such an approach using patient-derived iPSC seems a splendid solution for these issues, it is still costly and time-consuming to guarantee the quality and safety of iPSC and its derivative therapeutic cells; therefore, the project has launched to establish well-qualified “off-the-shelf” iPSC stock [8, 9]. The original aim of the iPSC stock project, led by the Center for iPS Cell Research and Application, CiRA, is to stock HLA-typed iPSCs. In order to reduce the risk of immune rejection, HLA-homozygous individuals were thought to be optimal for iPSC donors. Scientists estimated that banking of approximately 140 unique HLA haplotypes could cover 90% of the Japanese population [10]. According to the Japanese HLA database in HLA laboratories, screening of 160,000 individuals is required to find 140 unique clones. Since its foundation, the project has established 27 iPSC lines from 7 donors of 4 HLA-homozygous haplotypes, which cover approximately 40% of Japanese HLA haplotypes. These lines are currently available and distributed from CiRA Foundation for research use. Taylor et al. also estimated that 150 homozygous HLA-haplotypes match 93% of the UK population [9]. In Korea, it has been estimated that the top 20 ranked HLA-haplotypes may cover more than 80% of their population [11]. Because Japanese and Korean peoples are genetically closely related, 5 out of the top 10 haplotypes are common in these populations. Not only nationwide but also a worldwide collaboration would accelerate the construction of HLA-typed iPSC bank.

As described above, the stock project of HLA-homozygous iPSC has been launched with high expectations; however, recent accumulating evidence suggests that this approach may not entirely prevent the immune rejection of iPSC-derived transplants.

## iPSC-based transplantation and immune reaction

Immune rejection of transplanted allografts is often caused by a genetic mismatch of major histocompatibility complex (MHC) and following recipient T cell activation [12]. The vast diversity of MHC haplotype in humans makes it arduous to find MHC fully matched donor and recipient pairs, except for identical twins. If the immune rejection is caused only by HLA (human MHC molecule) mismatch, HLA-matched and HLA-haplotype homozygous to heterozygous transplantation has a minimum risk of rejection.

Given that the “iPSC stock project” intends to prepare HLA-haplotype homozygous clones, the possibility of matching with recipients’ HLA may increase. However, it should be noted that genetic mismatches of non-MHC molecules, so-called minor antigens, also provoke a strong immune rejection despite the MHC matched donor-recipient combination [12, 13]. Steers et al. reported one recent example that antigen mismatch due to gene-disrupting mutation in LIMS1 gene [14]. They found that the recipient’s inheritance of variants that disrupt the expression of LIMS1 in the kidney urges the recipient to allosensitization and rejection.

In accordance with previous findings, practical transplantation models of HLA-homo iPSC also demonstrated the risk of immune rejection [15, 16]. Kawamura et al. generated cardiomyocytes from cynomolgus macaque iPSC (iPSC-CMs), whose MHC-haplotype was homozygous, and assessed their survival in MHC-matched or MHC-mismatched allogeneic recipients. The combination of tacrolimus, mycophenolate mofetil, and prednisolone showed their therapeutic effect in MHC-matched recipients that results in iPSC-CMs survival for 2 months. This combination therapy was also effective in two out of three MHC-mismatched recipients when administered at the same concentration as in the MHC-matched group. It is noteworthy that reduced immunosuppression was not capable of maintaining iPSC-CMs survival in MHC-matched recipients, and that without any immunosuppressant, iPSC-CMs scarcely survived for 1 month. As these results indicate, matching iPSC-MHC and recipient-MHC allow for the long-term survival of grafts in particular with immunosuppression at a sufficient level even for MHC-mismatched recipients. A similar result was also described in iPSC-derived striatal neuron injection to Huntington’s disease model of cynomolgus monkey [17]. Badin et al. demonstrated that matching MHC haplotype between donor and recipient was not sufficient to avoid immune rejection in the experimental model. Although we need to be aware that striatal neurons have distinct immunogenic profiles from dopaminergic neurons, which was previously reported successful engraftment [18], this finding depicts a potential risk for rejection of MHC-matched allografts.

Allogeneic grafts are targeted not only by T cells but also by NK cells [19]. T cells acquire restriction to self-MHC through their development [20]. Similar to this fundamental characteristic of T cells, NK cells recognize self MHC and attack target cells that fail to express sufficient MHC molecules [21, 22]. This principle of NK cell action, known as “missing-self,” elicits an immune reaction in MHC homo-to-hetero transplantation. NK cells randomly express their activation and inhibitory receptors [23]; in human, KIR2DL1 and KIR2DL3 receive inhibitory signals from C1 and C2 type of HLA-C, respectively [24]. In the setting of HLA-C1 or C2 homo-to-C1/C2-hetero transplantation, donor cells express either C1 or C2 type HLA-C and could only suppress corresponding KIR2DL1 or KIR2DL3 expressing NK cells. Thus, C1-homo grafts will be rejected by KIR2DL3-NK cells, and C2-homo grafts by KIR2DL1-NK cells. In this regard, Ichise et al. demonstrated that HLA-hetero recipient NK cells reject T cells and vascular endothelial cells derived from HLA-homo iPSC [16]. They showed that transduction of type 2 HLA-C gene into type 1 HLA-C homozygous iPSC rescued iPSC-derived vascular endothelial cells from specific lysis by HLA-hetero NK cells. These results showed the evidence that HLA-homo transplants can be potentially targeted by recipient NK cells in the HLA-homo iPSC stock model.

Antibody-mediated rejection (AMR) has been another critical issue in organ transplantation [25]. It is well-known that anti-MHC antibody is involved in AMR [26, 27], and thus, MHC-matched transplantation can reduce the risk of AMR. However, antibody production can be induced against non-MHC molecules [28, 29]. Considering HLA-homo iPSC to HLA-hetero recipient transplantation, anti-MHC antibody production may be prevented, whereas anti-non-MHC antibody is still possibly induced and causes AMR. Once AMR is initiated, current therapeutic regimens have limited effects on its regulation [30, 31]. Hence, similar to organ transplantation, it is also of great importance to block anti-donor antibody production in HLA-homo iPSC-based therapy.

## Complications with conventional immunosuppressant therapy

A number of immunosuppressants have developed after decades of endeavors, including low-molecular-weight compounds (e.g., calcineurin and mTOR inhibitors) and molecular target drugs (e.g., anti-CD3 or anti-CD25) [32]. However, continuous use of immunosuppressants may result in several side effects caused by systemic immune suppression [33]. Ultimately, when there is a need to discontinue immune suppression, it leads to the loss of transplanted grafts. Tolerance of a transplanted organ is considered as one possible way to circumvent this problem. Clinically, transferring donor bone marrow or donor hematopoietic stem cells have achieved long-term allograft survival without maintenance immunosuppression [34–36]. Besides, other attempts of transplant tolerance have been reported by means of transferring regulatory T cells (Treg), regulatory macrophages, or anergic T cells [37–39].

Transplantation can be divided into two modes; one is organ transplantation, and the other is tissue transplantation. It has been suggested that different allogeneic transplants have hierarchical susceptibility to rejection [40]. Delayed transfer of allogeneic MHC-specific T cells rejected pre-transplanted skin and islet allografts at a much smaller number of T cells than that was required for cardiac allograft rejection. This observation may be, in part, explained by the size of allograft, which determines the sensitivity to cytokine. Upon inflammation, interferon-γ would upregulate MHC expression by allografts leading to enhanced sensitivity to allo-MHC specific T cells. Besides, Fas ligand expression by vascular endothelial cells may also support the less rejection susceptibility of solid organ allografts. Indeed, endothelial cells modified to overexpress functional, cell-surface Fas ligand attenuated T cell and macrophage in a rat model of transplant graft vasculopathy [41]. Considering that iPSC-derived allografts are “tissues,” these iPSC-allografts will be suffered from severe rejection, and in some cases, patients need lifelong immunosuppressant therapy. Therefore, the aforementioned problems accompanying systemic immune suppression would be a hurdle for iPSC-based therapy to spread into our society.

## Gene modification to suppress immune activation

As outlined above, we need to take into account the immunosuppressive approach in the case of iPSC-based therapy. It is worth noting that ESCs are always allogeneic to recipients, so that immune regulation in ESC-based therapy has also been discussed. Recent research concerning this issue demonstrated human ESC-graft acceptance in allogeneic recipients without immunosuppressive medications.

**Fig. 1:**
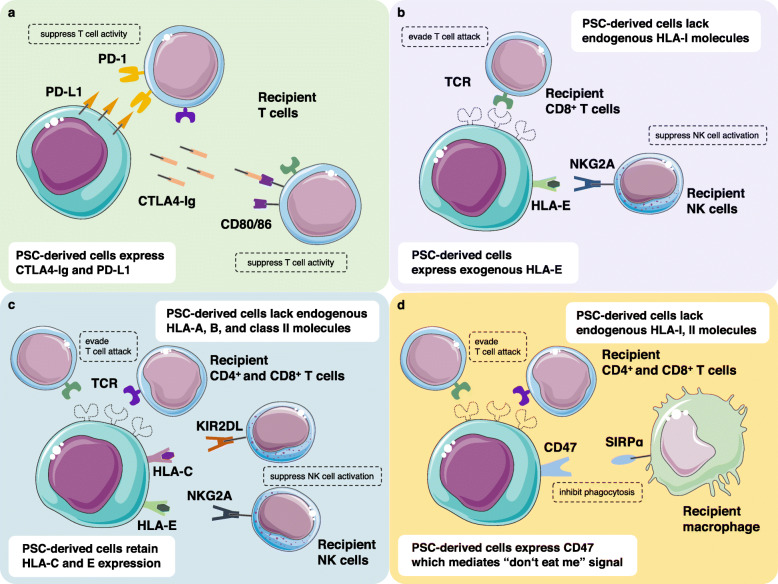
Genetic modification of pluripotent stem cells to suppress or evade recipient immune cell attack. **a** Gene transduction of CTLA4-Ig and PD-L1 to PSCs. **b** HLA-I gene knockout in combination with HLA-E gene transduction. **c** HLA-A, B, and class II gene knockout in combination with retained expression of endogenous HLA-C and E. **d** HLA-I and II gene knockout in combination with CD47 gene transduction

In the field of cancer immunology, immune checkpoint molecules are attracting attention to their significant roles in modulating immune reaction against cancer cells. Monoclonal antibodies targeting programmed death-1 (PD-1) and cytotoxic T-lymphocyte antigen 4 (CTLA4) are widely known drugs and have proven strikingly powerful in augmenting anti-cancer immunity led to the elimination of cancer cells [42–44]. These molecules are expressed on activated T cells to repress their proliferation and cytotoxicity, and prevent damages to the self [45–47]. In the view of the fact that regulating immune reaction to allografts is the primary purpose of taking immunosuppressive drugs, immune checkpoint molecules on the surface of anti-donor T cells would be valuable targets. Rong et al. from Shenzhen Children’s Hospital have addressed whether the genetic introduction of CTLA4 fused with the immunoglobulin domain (CTLA4-Ig) and programmed death ligand-1 (PD-L1) into human ESC, denoted CP hESCs, could protect them from attack by recipient T cells (Fig. 1a) [48]. Transgenes were inserted into hypoxanthine phosphoribosyltransferase-1 locus, which maintain expression after differentiation. CP hESCs were induced to differentiate to fibroblasts and cardiomyocytes and were transplanted to humanized NSG mice. Their results depicted that CP hESCs-derived grafts were infiltrated with a smaller ratio of CD3+ cells over human nuclear staining+ cells. Of importance, neither single transduction of PD-L1 nor CTLA4-Ig could protect CP hESCs; CP hESCs-derived teratoma was rejected entirely. Their approach has described the significance of targeting immune checkpoint molecules in ESC-based transplantation in a fully allogeneic donor-recipient combination, which would be followed by iPSC-based therapy.

## Gene modification to escape from the immune system

Allogeneic transplantation elicits T cell activation, which recognizes donor MHC as non-self. Thus, a straightforward method is erasing MHC expression from donor cell surface. In this case, recipient T cells would ignore donor cells and not be activated by a genetic difference of MHC. Some groups have previously addressed the efficacy of this approach [49, 50]; however, in turn, NK cells will exert a cytotoxic effect on MHC-null allografts [51, 52]. In addition to KIRs, NK cells express NKG2A as an inhibitory receptor [53]. The ligand for NKG2A in humans is HLA-E that has a limited polymorphism compared with other HLA molecules. Gornalusse et al. have attempted to achieve allograft survival of human ESCs that are expressing transduced HLA-E but have been deleted for endogenous class I type HLA molecules (Fig. 1b) [54]. The research group genetically deleted beta-2 microglobulin, a key molecule consisting of MHC class I, by knock-in exogenous HLA-E gene. Their in vitro analysis demonstrated that genetically engineered ESC-derived CD45 positive cells escaped from specific lysis by NK cells while neutralizing antibody to HLA-E or NKG2A restored cytotoxicity of NK cells. Besides, pre-formed teratoma of HLA-E knock-in human ESC in immunodeficient mice was not rejected by transferring CD8+ T cells primed with HLA expressing embryoid bodies. As HLA-E can only suppress NK cells expressing NKG2A, KIR2DL1, 2, 3, and 4 expressing NK cells still retain potential to attack HLA-E expressing allograft. Xu et al. introduced similar approach through directly targeted disruption of HLA-A, B, and class II molecules but not HLA-C and E in human iPSC (Fig. 1c) [55]. In addition to HLA-E interaction with NKG2A, HLA-C binding to KIR2DL molecules mediates suppression signal to recipient NK cells. Hematopoietic cells derived from those HLA-C/E-expressing but A/B-KO iPS cells could evade both CD8+ T cell and NK cell killing. In vivo bioluminescent assay also demonstrated the engineered cell’s potential to survive in allogeneic transplantation. What described above are the probable approaches to evade host immune responses by engineering donor cells. Likewise, the hypoimmunogenic PSC was established by the genetic ablation of MHC molecules along with introducing CD47, a cell surface molecule that interacts with its receptor, SIRPa, to inhibit phagocytosis (Fig. 1d) [56]. The idea of this universally compatible stem cells by Deuse et al. was brought by maternal tolerance to fetal tissue. They found that syncytiotrophoblast cells that divide the maternal and fetal side of the bloodstream are expressing low MHC class I and II molecules but a high amount of CD47. They form placental immune privilege through their extensive potential of suppressing immune activation. Deuse et al. generated endothelial cells, smooth muscle cells, and cardiomyocytes from the modified iPSCs and assessed whether the cells could evade from immune cell attack, and as a result, sustained luminescent signals of the iPSC-derived grafts proved their survival in allogeneic transplantation models.

As a donor, PSC allows us to genetically modify its immunogenicity, which is not possible for conventional transplantation. Thus, the above attempts to establish hypoimmunogenic donor cells will develop a fundamental framework for future PCS-derived allograft transplantation.

## Novel cellular treatment for PSC-based transplantation

A number of attempts have tried cellular treatment for allograft survival. Generally, cellular therapy to rejection was mainly conducted by the use of donor cells. Within the context of PSC-based therapy, that one donor provides numerous chances of transplantation, repeated collection of donor cells is not clinically feasible. However, taking advantage of the feature that PSCs have differentiation potential into any cells, including ones with immune regulatory function, cellular treatment for allograft survival may be a possible approach even in case of PSC-based therapy.

Cai et al. from the National Research Institute for Child Health and Development, Japan, generated regulatory dendritic cells from mouse iPSC (iPS-DCreg) and treated cardiac transplantation recipients with the regulatory cells [57]. Recipient mice showed an increased proportion of Treg in the spleen and activated GITR^+^ Treg in cardiac allografts, and as a result, iPS-DCreg administration exhibited its capability of donor-specific allograft acceptance. The research team employed a cardiac transplantation model and demonstrated the possibility of iPS-DCreg therapy, which makes us expect a further attempt to regulate immune reaction to iPSC-derived tissue allograft.

**Fig. 2:**
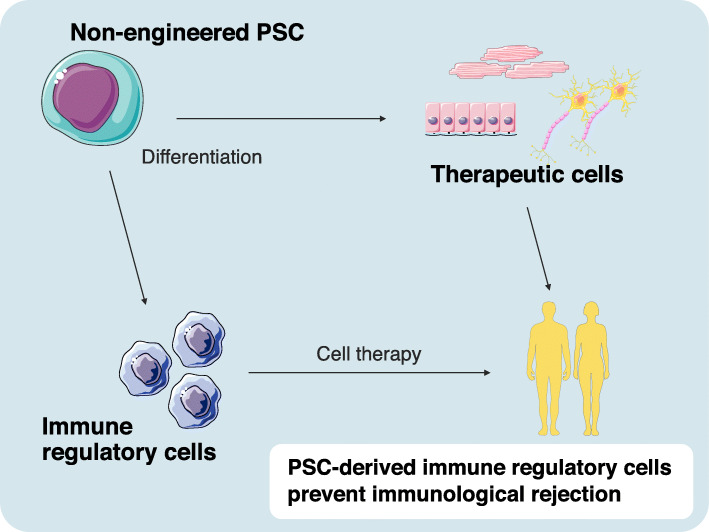
Immunoregulatory cell induction from pluripotent stem cells. Immunoregulatory cell differentiation and combined cell therapy to prevent immunological rejection against PSC-derived therapeutic cells

We recently proposed a novel concept of cell therapy with the goal of immune regulation in the era of regenerative medicine (Fig. 2). A hallmark feature of our experimental model is the regulation of recipient immune response against PSC-derived allograft by the administration of “the same PSC-derived” immunoregulatory cells [58, 59]. To verify the concept, we developed a therapeutic model of PSC-derived immunosuppressive cells.

## Generation of immune regulatory cells to prevent immunological rejection

As a transplantation model, we prepared an allogeneic pair of iPSC (BALB/c) and recipients (C3H). Donor iPSCs were stimulated with a stepwise combination of several growth factors for a total of 24 days. Resulting cells expressed CD11b, F4/80, CD206, and CD14; these are known for macrophage markers. Also, these cells expressed the genes of immunosuppressive molecules such as Arginase1 and Nos2 [60]. Therefore, we expected their immunosuppressive function and denoted them as iPSC-derived macrophage-like suppressor cells (iPS-SCs). To assess their immunosuppressive function, we performed in vitro and in vivo assays. When we added iPS-SCs to a mixed culture of BALB/c DC and C3H T cells, the percentage of proliferated T cells was significantly lower than a mixed culture condition in the absence of iPS-SCs. Moreover, the iPSC-SCs immunosuppressive function was canceled by the addition of inducible nitric oxide synthase (iNOS, coded by Nos2) inhibitor, indicating that reduced T cell proliferation by iPS-SC supplementation was dependent on iNOS. We next generated cardiomyocytes from donor iPSC line as tissue allograft. Cardiomyocytes were induced from BALB/c iPSC and transplanted into C3H kidney subcapsular. As a result, we observed rapid rejection in the non-treatment group (6–12 days) while allograft survival was significantly prolonged in the iPS-SC treatment group even without any immunosuppressive drugs (14–28 days). To our surprise, iPS-SC injection reduced the serum level of the anti-donor antibody. This observation indicates that iPS-SC treatment may prevent anti-donor antibody production and reduce the risk of AMR. This research was the first report that attempted to prevent rejection for iPSC-derived allograft by means of the treatment with iPSC-derived immunoregulatory cells [61].

In order to extend our concept, we challenged to regulate the recipient’s immune system by another approach. For recipient immune regulation, we focus on the thymus. Immunological rejection is mainly mediated by T cells that develop and differentiate within the thymus. The thymus plays a significant role in the establishment of immunological self-tolerance. Thymic epithelial cells (TECs), consisting of the thymus epithelium, largely contribute to this system by their high ability of self-antigen expression and presentation [62]. Previous studies revealed that host immune reaction to allogeneic transplants could be regulated by transplantation of thymus epithelial tissue that results in the deletion of donor reactive T cells or Treg generation [63–66]. So we aimed to generate TECs from iPSC, but former attempts on the generation of TECs by conventional in vitro culture raised the problem on its induction efficiency [67–70]. In the study, we transduced transcription factor *Foxn1* into iPSC and induced differentiation according to our stepwise in vitro culture protocol [71]. In addition, we assessed whether iPSC-derived TEC-like cells (iPSC-TEC) could function as immune-regulatory cells in iPSC-based therapy. Our analysis demonstrated that Foxn1-expressing iPSC differentiated to TEC-like cells more efficiently than the Foxn1 non-transduced cell line. Furthermore, we evaluated the contribution of iPSC-TECs to the allograft survival in the transplantation model. Recipient C3129F1 mice received B6 iPSC-TEC or control fibroblast transplantation under the left and right subcapsular renal space by following anti-CD4 and CD8 antibody treatment and 3 Gy total body irradiation. Control recipients rejected B6 and BALB/c skins; on the other hand, recipients of B6 iPSC-TEC showed significantly prolonged survival of B6 skin. In this group, skins from BALB/c were rejected independently of pre-transplanted iPSC-TEC. Overall, these results suggested that iPSC-TEC would contribute to graft survival, specifically when its genetic background is identical with iPSC-TEC.

From the series of experiments, we delineated a novel therapeutic possibility for PSC-based transplantation that generates not only therapeutic graft but also immune-regulating cells from established PSC lines.

## Conclusion

As the number of researches about PSC-based therapy is increasing, immunological concerns are attracting more attention. Researchers have proposed several possible approaches that (i) suppress immune cell activation through genetic transduction of co-inhibitory molecules [48], (ii) erase or compensate histocompatibility antigens with the intend to hide PSC-allografts from recipients immune surveillance [54–56], and (iii) induce immunoregulatory cells from donor PSCs to protect PSC-allografts from immunological rejection [58, 59, 71]. Genetic depletion of MHC in combination with the compensation of inhibitory molecules is an innovative approach. However, as they mainly focused on preventing T cell or NK cell-mediated rejection, antibody production by B cells is still a significant threat to allograft. Moreover, because donor antigens are presented to T cells not only by conventional antigen-presenting cells but also by recipient vascular endothelial cells that feed allografts [72], solely modifying donor cells may not achieve long-term engraftment. Since hypoimmunogenic cells escape recipient immune surveillance, we need to thoroughly consider tumorigenicity and the way of clearance of the cells upon infection. In this regard, not evading immune attack, but inducing an immune tolerance may be an attractive approach. Including our group, several attempts aimed to induce transplant tolerance in PSC-based therapy. Still, to date, significant issues on the optimal cell types, therapy regimen, duration of its effect, and so on remain to be addressed. In any approach, gene transduction always comes with the possibility of gene silencing. Taken together, although we are still on the way to establish bona fide practical methods, recent efforts certainly made step-by-step improvements.

PSC-based technologies bring us a chance to touch many clinical options, which have never been thought about before. It will now be important to consider the safety of this novel technique from an immunological point of view and that supports successful clinical translation of PSC-based cell therapy.

## Data Availability

Not applicable

## Abbreviations

AMR: Antibody-mediated rejection
CTLA4: Cytotoxic T-lymphocyte antigen 4
CTLA4-Ig: CTLA4 fused with the immunoglobulin domain
HLA: Human leukocyte antigen
iNOS: Inducible nitric oxide synthase
iPSC: Induced pluripotent stem cell
iPS-Dcreg: Regulatory dendritic cells from mouse iPSC
iPS-SC: iPSC-derived macrophage-like suppressor cells
iPSC-CM: Cardiomyocytes from cynomolgus macaque iPSC
MHC: Major histocompatibility complex
PD-1: Programmed death-1
PD-L1: Programmed death ligand-1
PSC: Pluripotent stem cell
TEC: Thymic epithelial cell
Treg: Regulatory T cell
